# Healthcare Workers' Perception and Compliance on Personal Protective Equipment Use in Critical Care Units of a Tertiary Care Hospital in Bahrain

**DOI:** 10.7759/cureus.69106

**Published:** 2024-09-10

**Authors:** Athraa S Naser, Tamer Abo Arisheh, Rommel Acunin, Harold C Cabanalan, Safa Alkhawaja, Azhar M Salman, Zainab T Khamdan, Fatima A Durazi

**Affiliations:** 1 Infection Prevention and Control, Government Hospitals Bahrain, Manama, BHR

**Keywords:** compliance, hcw, perception, ppe, salmaniya medical complex

## Abstract

Introduction

Contact precautions or the use of personal protective equipment (PPE), specifically gowns and gloves, has become a common practice in intensive care units (ICUs) as part of robust infection prevention and control efforts. Hence, a positive attitude toward PPE used among healthcare workers (HCWs) is critical to prevent healthcare-associated infections (HAIs).

Aim

This study aimed to determine the perception and compliance of HCWs working at critical care units regarding PPE used at government hospitals (GHs) in Bahrain.

Subject and methods

A cross-sectional study was conducted among HCWs working in critical units at GHs, Bahrain. A self-administered questionnaire consisting of three sections was sent to the targeted HCWs. The questionnaire consisted of socio-demographic characteristics (i.e., age, gender, job category, etc.), previous participation in PPE training, and a 15-item questionnaire to assess the perception and compliance of HCWs regarding PPE use.

Results

Among the 119 HCWs enrolled, 73.1% were females, and 48.7% were between 31 and 40 years old. The results of this study suggested that although 95.8% of participants received education about PPE use, only 48.7% had a good perception of PPE, and an even smaller percentage (12.6%) demonstrated good compliance. A positive, highly statistically significant correlation was found between perception and compliance scores (p<0.001). Increased perception and compliance scores were associated with increasing age, non-Bahrainis, and years of experience. Interestingly, doctors were more likely to demonstrate lower perception and compliance scores toward PPE use.

Conclusion

The perception of HCWs regarding PPE use was adequate, but their actual adherence was unsatisfactory. However, doctors' perceptions and compliance with PPE use were significantly lower than other HCWs. Further larger studies are required to establish the level of perception and compliance among HCWs in our region.

## Introduction

Contact precautions or the use of personal protective equipment (PPE), specifically gowns and gloves, has become a common practice in intensive care units as part of robust infection prevention and control efforts [1]. The United States Center for Disease Control and Prevention (US-CDC) recommends that for patients under contact precaution, healthcare workers (HCWs) must wear the recommended PPE upon entry and discard it before exiting the patient room to prevent infectious agents from spreading [2]. In many intensive care settings, universal use of gowns and gloves has been adopted as a standard protocol [3,4].

In government hospitals (GHs) in Bahrain, it is an institutional policy to screen patients for multidrug-resistant organisms (MDRO) upon admission to critical care units. Patients are placed on contact precaution while waiting for screening culture results or will continue the isolation if MDRO is detected. As the number of patients requiring contact precaution mounts, the burden on HCWs to perform their duties significantly increases [5]. The overall quality of patient care is also being affected. HCWs are less likely to visit their patients due to PPE donning and doffing inconvenience [6].

Limited literature has tackled HCWs' perceptions of and compliance with PPE use in the Kingdom of Bahrain. This was supported by a study conducted by Sowar Sr et al. (2023) regarding HCWs' hand hygiene (HH) knowledge and perception in a tertiary care hospital in Bahrain [7]. However, there are numerous similar published cross-sectional studies from neighboring countries [8-10], as well as from Asia Pacific [11] and the Western Region [12]. Most of these studies have explored the different experiences of HCWs regarding PPE use at the peak of the COVID-19 pandemic.

There are many factors affecting HCWs' compliance with PPE use. Staff education is perceived to be one of the most significant predictors of good compliance [13]. The provision of adequate training on proper use of PPEs in preventing transmission of infectious agents is one of the most effective parameters of HCWs adherence [14-17]. Continuous availability of PPE supplies has also been implicated as one of the major challenges faced by many organizations globally [17,18].

The fear of infection acquisition also influences HCWs adherence. In one study conducted by Abed Allah et al. (2021), HCWs perceived that the COVID-19 pandemic significantly increased their compliance with the use of PPEs as compared to pre-pandemic (p<0.001) [18,19]. HCWs' attitudes and behavior are shown to be positively impacted by their level of perceived risks [20,21]. Staffing issues and workload have also been stressed as one of the reasons that lead to poor compliance [11,18,22]. Another factor is staff comfort while on PPEs. In a scoping review conducted in Japan, the most frequently reported adverse events include headaches, heat, skin abrasions, and breathing discomfort [23]. This study is consistent with the findings of similar studies conducted in Saudi Arabia and Australia recently [24,25].

To the best of our knowledge, no study has ever been published in Bahrain regarding HCWs' perception and compliance with PPE use. Thus, our study explores the HCWs' insights and motivations for good compliance while working in a stressful and physically demanding environment such as critical care units.

## Materials and methods

This cross-sectional study was carried out among HCWs working in critical units at GH, Bahrain. Data were collected between March and May 2024 using Google Survey Form (GSF). GSF has been utilized to collect data from HCWs as it is free and due to the widespread use of Google accounts. A self-administered questionnaire consisting of three sections was distributed among HCWs, such as socio-demographics characteristics (i.e., age, gender, job category, etc.), previous participation with PPE training, and a 15-item questionnaire to assess the perception and compliance of HCWs regarding PPE use (see Appendix 1, Table 5 and Appendix 2, Table 6). Participants were informed of the voluntary nature of this study and provided informed consent prior to filling out the questionnaire. The survey form sent to participants was anonymous, and responses received were stored in a protected computer, and only a research team member was given access to ensure data transparency and credibility. All HCWs working in critical care units, aged between 20 and 60 years old, were recruited. Non-HCWs, non-critical care staff, and part-time or temporary contract employees were excluded from the final data analysis. The Research Committee for Government Hospitals has approved this project. Research approval serial no. 62-230524, on 23 May 2024.

Study area/setting

The study was conducted in one of the largest Government Hospitals in Bahrain, established in 1957. It is a multi-specialty care hospital with a capacity of more than 1500 beds, serving both locals and expatriates. It has a variety of critical care units for adults, pediatrics, neonates, and patients with coronary and burn injuries [26].

Sample size and sampling technique

Based on the Raosoft sample size calculator software [27], with the estimation of around 300 overall population size of HCWs of critical care staff working in GHs, Bahrain, with a confidence level of 95% and a margin of error of 5%, the calculated sample size was 169. A convenience sampling technique was applied to gather data among HCWs working in the critical care units until we reached the target sample size.

Pilot study

To ensure the validity of our questionnaire, we conducted a pilot study among 20 HCWs (11% of the total population) to determine the internal consistency of the study questionnaire. The questionnaire consisted of two domains: perception (seven items) and compliance (eight items). Both perception and compliance questionnaire (eight items) have categories ranging from "strongly disagree" coded with 1 to "strongly agree" coded with 5. The reliability test of the perception questionnaire has a Cronbach Alpha of 0.805 or 80.5%, indicating very good internal consistency, while the reliability test of the compliance questionnaire has a Cronbach Alpha of 0.788 or 78.8%, suggesting good internal consistency. The overall reliability test of the two questionnaires, which consisted of 15 items, had a Cronbach Alpha of 0.797 or 79.7%, indicating good internal consistency of the study questionnaire. Thus, the questionnaire was valid to use in this study.

Questionnaire criteria

The questionnaire was taken from previously published references, and the most appropriate items were selected and used in our survey as applicable to our hospital settings [13,16,19]. The perception of HCWs about PPE use has been assessed using a seven-item questionnaire, with five-point Likert scale categories ranging from "strongly disagree" coded with 1 to "strongly agree" coded with 5. Negative questions have been re-coded reversely to avoid bias in the score. The total perception score has been calculated by adding all seven items. A score ranging from seven to 35 points has been achieved. The higher the score, the higher the perception toward PPE used. By using 50% and 75% as cutoff points to determine the perception levels, respondents were considered as having poor perception if the total score was <50%, 50% to 75% were considered moderate, and above 75% were considered as having good perception levels [7].

Similarly, the compliance of HCWs about PPE use has been measured using an eight-item questionnaire, with five-point Likert scale categories ranging from "strongly disagree" coded with 1 to "strongly agree" coded with 5. Negative questions have been re-coded reversely to avoid bias in the score. Summing up eight items yielded a maximum score of 40, and the minimum score was eight points. The greater the score, the greater compliance toward PPE use. Similar criteria were applied to classify compliance scores into poor level (score <50%), moderate (score 50%-75%), and good compliance level (score >75%) [7].

Statistical analysis

Categorical variables were described as counts and proportions (%), while continuous variables were computed and expressed as means and standard deviations. The differences in the perception and compliance scores in relation to HCWs' socio-demographic characteristics have been conducted using the Mann-Whitney Z-test and Kruskal Wallis H-test. Post-hoc analysis was also performed to determine the multiple mean differences between perception and compliance scores in relation to job categories using the Dunn-Bonferroni test (post-hoc analysis for the non-normally distributed data). The normality test was evaluated using the Kolmogorov-Smirnov test (for sample size >50). The perception and compliance scores follow the non-normal distribution. Thus, the non-parametric tests were applied. The Spearman correlation coefficient has been performed to determine the correlation between perception and compliance scores. A p-value of less than 0.05 was considered statistically significant for all statistical tests. All statistical data were analyzed using Statistical Packages for Social Sciences (SPSS) version 26 (IBM Corp., Armonk, NY, USA).

## Results

We sent the questionnaire among the targeted HCWs through GFS to collect the required sample size (N=269), and one hundred and nineteen HCWs responded (response rate: 70.4%). We did not achieve the required sample size as some critical care HCWs were on leave or had incorrect email information. Table 1 describes the socio-demographic characteristics of HCWs. About 48.7% were between 31 and 40 years old, with females being dominant (73.1%). The majority (68.9%) were non-Bahrainis. Nurses constitute most HCWs (73.9%), and more than half (50.4%) had over ten years of working experience. Nearly all received education about PPE use (95.8%), whereas 77.2% received PPE education within GH premises.

**Table 1:** Socio-demographic characteristics of HCWs (n=119). *Variable with multiple response answers. HCWs: healthcare workers, SMC: Salmaniya Medical Complex, PPE: personal protective equipment.

Study variables	N (%)
Age group	
20–30 years	20 (16.8%)
31–40 years	58 (48.7%)
41–50 years	35 (29.4%)
>50 years	06 (05.0%)
Gender	
Male	32 (26.9%)
Female	87 (73.1%)
Nationality	
Bahraini	37 (31.1%)
Non-Bahraini	82 (68.9%)
Job category	
Doctor	13 (10.9%)
Nurse	88 (73.9%)
Physiotherapist	04 (03.4%)
Respiratory therapist	14 (11.8%)
Years of experience	
<1 year	05 (04.2%)
1-5 years	28 (23.5%)
6-10 years	26 (21.8%)
>10 years	60 (50.4%)
Received education about PPE use	
Yes	114 (95.8%)
No	05 (04.2%)
Specific institutions that provided PPE education (n=114)*	
College	26 (22.8%)
Previous hospital	34 (29.8%)
SMC	88 (77.2%)

**Figure 1:**
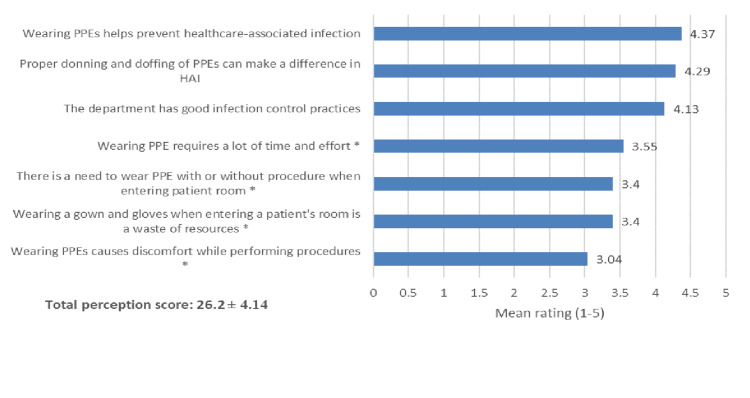
Assessment of perception toward PPE used. *Reverse coded items. Response has a range from "strongly disagree" coded with 1 to "strongly agree" coded with 5. PPE: personal protective equipment, HAIs: healthcare-associated infections.

Regarding the assessment of perception toward PPE use (Figure 1), it was observed that the top three statements with the highest ratings were "Wearing PPEs helps prevent healthcare-associated infection" (mean score: 4.37), followed by "Proper donning and doffing of PPEs can make a difference in HAI" (mean score: 4.29) and "The department has good infection control (IC) practices" (mean score: 4.13), while statement "Wearing PPEs causes discomfort while performing procedures" (mean score: 3.04) obtained the lowest rating. The total mean score of the perception questionnaire was 26.2 (SD 4.14).

**Figure 2:**
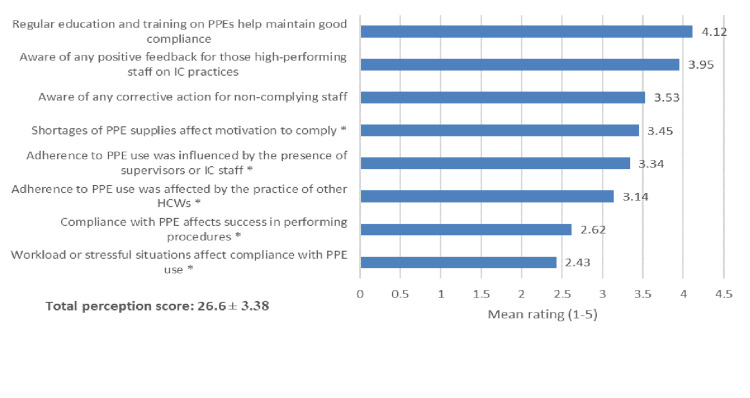
Assessment of compliance toward PPE used. *Reverse coded items. Response has a range from "strongly disagree" coded with 1 to "strongly agree" coded with 5. PPE: personal protective equipment, HCWs: healthcare workers, IC: infection control.

Regarding the assessment of compliance toward PPE use, the top three statements with the highest ratings were "Regular education and training on PPEs help maintain good compliance" (mean score: 4.12), "Aware of any positive feedback for those high-performing staff on IC practices" (mean score: 3.95), and "Aware of any corrective action for non-complying staff" (mean score: 3.53), while statement "Workload or stressful situations affect compliance with PPE use" achieved the lowest rating (mean score: 2.43). The overall mean compliance score was 26.6 (SD 3.38) (Figure 2).

**Figure 3:**
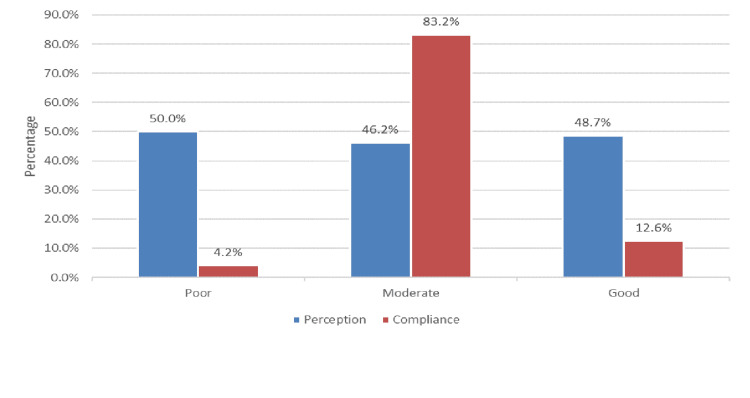
Level of perception and compliance toward PPE used. PPE: personal protective equipment.

Figure 3 shows the level of perception and compliance toward PPE used. It was revealed that poor and moderate perception levels constituted 5%, 46.2%, and 48.7%, respectively. Also, poor, moderate, and good compliance levels were found in 4.2%, 83.2%, and 12.6%, respectively.

**Figure 4:**
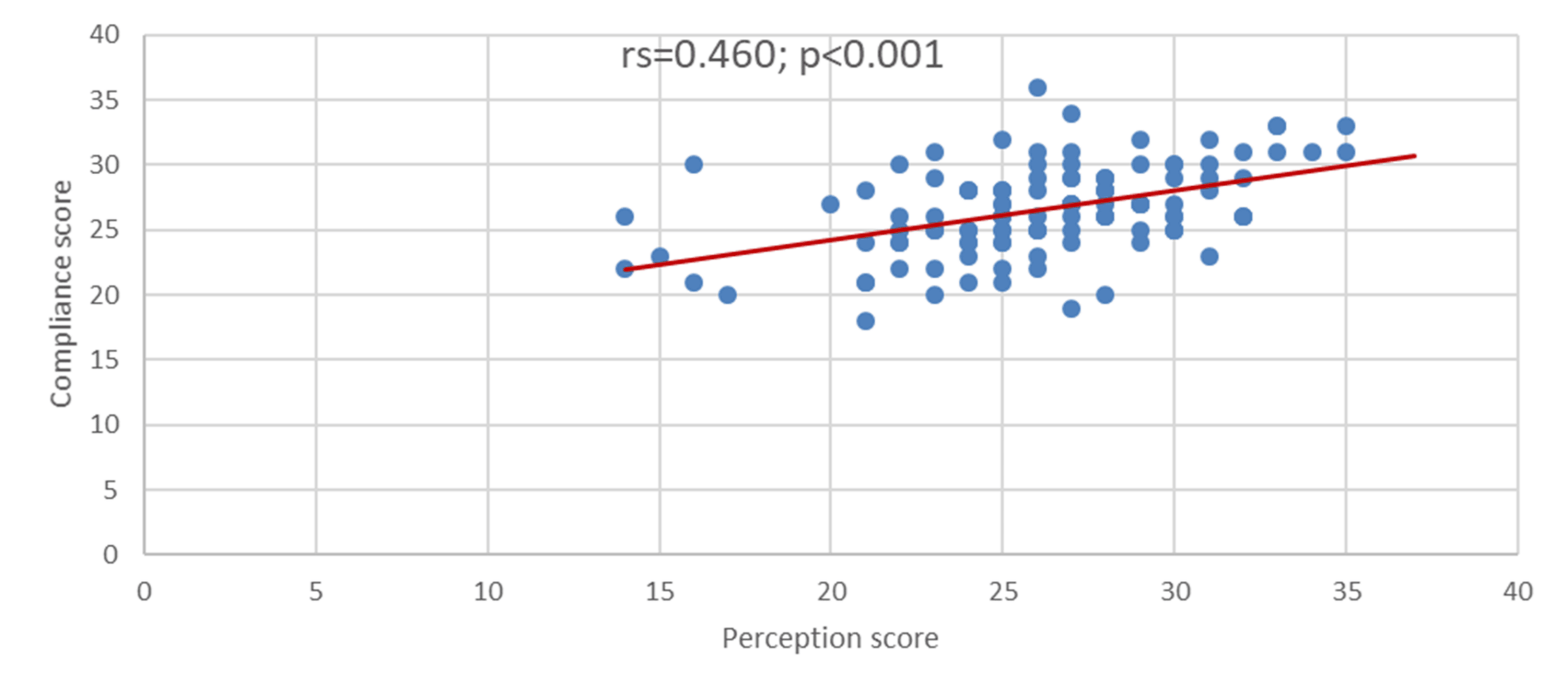
Correlation between perception and compliance scores.

Figure 4 shows a positive significant correlation between perception and compliance scores (rs=0.460; p<0.001). Suggesting that the increase in the score of compliance correlates with the increase in the score of perception.

Measuring the differences between perception score and the socio-demographic characteristics of HCWs found that a higher perception score was more associated with increasing age (Z=3.319; p=0.001), non-Bahraini (Z=4.165; p<0.001) and increasing years of experience (Z=2.517; p=0.012), while lower perception score was more associated with being a doctor (H=12.379; p=0.002). No significant differences were observed between the perception scores in terms of gender, received PPE education during college, and previous employment at different hospitals or GH (p>0.05) (Table 2).

**Table 2:** Differences in perception score in relation to the socio-demographic characteristics of HCWs (n=119). Results are expressed as mean ± standard deviation (SD). HCWs: healthcare workers, SMC: Salmaniya Medical Complex. §P-value has been calculated using Mann-Whitney Z-test. ‡P-value has been calculated using Kruskal-Wallis H-test. **Significant at p<0.05 level.

Factor	Perception score (35), Mean ± SD	Z-test	P-value^§^
Age group			
≤40 years	25.3 ± 3.96	3.319	0.001**
>40 years	27.8 ± 4.03		
Gender			
Male	25.4 ± 4.43	0.920	0.357
Female	26.5 ± 4.02		
Nationality			
Bahraini	23.9 ± 3.97	4.165	<0.001 **
Non-Bahraini	27.2 ± 3.80		
Job category^‡^			
Doctor	21.8 ± 4.52	12.379	0.002**
Nurse	26.8 ± 3.78		
Physio/Respiratory therapists	26.5 ± 3.93		
Years of experience			
≤10 years	25.2 ± 4.21	2.517	0.012**
>10 years	27.2 ± 3.87		
Received PPE education during college			
Yes	26.2 ± 3.32	0.148	0.882
No	26.2 ± 4.36		
Received PPE education from previous hospital employment			
Yes	27.1 ± 3.39	1.251	0.211
No	25.8 ± 4.36		
Received PPE education at SMC			
Yes	26.4 ± 4.18	0.629	0.529
No	25.7 ± 4.05		

When exploring the association between the compliance score and the socio-demographic characteristics of the HCWs (Table 3), we found that a higher compliance score was more associated with increasing age (Z=3.365; p=0.001), non-Bahraini (Z=2.820; p=0.005), increasing years of experience (Z=2.747; p=0.006) and received PPE education from SMC (Z=2.299; p=0.021). In contrast, a lower compliance score was more associated with being a doctor (H=12.481; p=0.002). No significant associations were observed between the compliance scores in terms of gender, received PPE education during college, and previous employment at different hospitals (p>0.05).

**Table 3:** Differences in compliance score in relation to the socio-demographic characteristics of HCWs. Results are expressed as mean ± standard deviation (SD). HCWs: healthcare workers, PPE: personal protective equipment, SMC: Salmaniya Medical Complex. §P-value has been calculated using Mann-Whitney Z-test. ‡ P-value has been calculated using Kruskal Wallis H-test. **Significant at p<0.05 level.

Factor	Compliance score (40), Mean ± SD	Z-test	P-value^§^
Age group			
≤40 years	25.8 ± 3.05	3.365	0.001**
>40 years	27.9 ± 3.57		
Gender			
Male	26.1 ± 3.44	0.587	0.557
Female	26.7 ± 3.36		
Nationality			
Bahraini	25.2 ± 3.74	2.820	0.005**
Non-Bahraini	27.2 ± 3.04		
Job category^‡^			
Doctor	23.2 ± 3.11	12.481	0.002**
Nurse	26.9 ± 3.18		
Physio/respiratory therapists	27.2 ± 3.28		
Years of experience			
≤10 years	25.7 ± 3.09	2.747	0.006**
>10 years	27.5 ± 3.43		
Received PPE education during college			
Yes	26.0 ± 3.29	0.746	0.456
No	26.7 ± 3.41		
Received PPE education from previous hospital employment			
Yes	26.2 ± 3.28	1.135	0.257
No	26.7 ± 3.43		
Received PPE education at SMC			
Yes	26.9 ± 3.45	2.299	0.021**
No	25.5 ± 2.94		

Post-hoc analysis was then performed to determine the multiple mean differences of perception and compliance scores in relation to job category (Table 4). The Dunn-Bonferroni test was employed to determine the differences in scores in the abnormal data distribution. Based on the results, it was revealed that there was a statistically significant difference in perception scores between doctor and nurse (p<0.001) and between doctor and physio/respiratory therapy (p=0.004). Further, we found a significant mean difference in compliance scores between doctor and nurse (p<0.001) and between doctor versus physio/respiratory therapy (p=0.002).

**Table 4:** Multiple mean differences of perception and compliance scores in terms of job category (n=119). Post-hoc analysis has been conducted using the Dunn-Bonferroni test. **The mean difference is significant at the p<0.05 level.

Dependent variable	(I) Job category	(J) Job category	Mean difference (I-J)	Std. error	Sig.	95% confidence interval	
						Lower bound	Upper bound
Total perception score	Doctor	Nurse	−4.90385^**^	1.15431	0.000	−7.7079	−2.0998
		Physio/respiratory therapists	−4.65385^**^	1.41400	0.004	−8.0887	−1.2190
	Nurse	Doctor	4.90385^**^	1.15431	0.000	2.0998	7.7079
		Physio/respiratory therapists	0.25000	1.00497	1.000	−2.1913	2.6913
	Physio/respiratory therapists	Doctor	4.65385^**^	1.41400	0.004	1.2190	8.0887
		Nurse	−0.25000	1.00497	1.000	−2.6913	2.1913
Total compliance score	Doctor	Nurse	−3.80070^**^	0.94688	0.000	−6.1008	−1.5006
		Physio/respiratory therapists	−4.01282^**^	1.15989	0.002	−6.8304	−1.1952
	Nurse	Doctor	3.80070^**^	0.94688	0.000	1.5006	6.1008
		Physio/respiratory therapists	−0.21212	0.82437	1.000	−2.2147	1.7904
	Physio/respiratory therapists	Doctor	4.01282^**^	1.15989	0.002	1.1952	6.8304
		Nurse	0.21212	0.82437	1.000	−1.7904	2.2147

Summary

The results of this study indicate that despite the good perception of HCWs toward PPE use, their actual adherence seems to be inadequate and, thus, needs more improvement. Doctors were shown to have the lower perspectives in terms of PPE use among all HCWs. Increasing years of experience and being a non-Bahraini were identified as the most important factors of increased perception and compliance. In addition, nearly all HCWs were able to attend education about PPE use.

## Discussion

This study examined the perception and compliance of HCWs working at critical care units regarding PPE use. Limited studies in Bahrain discussed the HCWs' perspectives on the appropriate use of PPE, except the study of Sowar et al. (2023); however, this study focused on HCWs' perception and knowledge of HH [7] but not of PPE in general. Hence, the findings of this study could be an essential contribution to the literature, given the high prevalence of HAIs in hospital settings, particularly in the event of an outbreak.

Level of perception

The level of HCWs' perception regarding PPE use was sufficient. According to the given criteria, about half of the respondents had good perception levels (49%), and only 5% achieved poor ratings (mean score: 26.2 out of 35 points). Among perception items, perception was highest toward the benefits of wearing PPEs in preventing HAI, followed by the appropriate sequence of donning and doffing of PPE and the belief that there is good infection control within the department. In contrast, the lowest perception was seen in discomfort with wearing PPEs while performing procedures on patients. This corroborates the findings of Abalkhail et al. (2021). Most HCWs (80%, 61.5%, and 73.2%) demonstrated good knowledge, attitude, and infection prevention practices [10]. Following these reports, Yusuf et al. (2023) indicated correct knowledge of proper donning and doffing was seen in almost half of HCWs [14]. Positive perception toward PPE use among HCWs is critical to prevent HAIs. Our HCWs seem to be on the right path of perception. However, given this scenario, continuous education should be promoted to maintain the knowledge of HCWs regarding the use of PPE in critical care settings within the hospital.

Significant factor of perception

The perception varies significantly by age, nationality, job categories, and years of experience. In particular, better perceptions were prevalent in the older age group (p=0.001), non-Bahrainis (p<0.001), and having more years in practice (p=0.012). This is strikingly similar to that of Sowar et al. (2023) [7]. Increasing age, non-Bahrainis, nurses, and increasing years of experience were associated with increased perception. Consistent with these reports, Abalkhail et al. (2021) found that HCWs' age and attendance at training were associated with good knowledge while increasing years of experience were associated with positive attitudes. However, the findings of our study suggest that gender, attendance on PPE education during college, previous hospital employment, and GH showed no significant association with perception (p>0.05). These non-significant associations could mainly be due to the limited sample size, necessitating further investigations.

Level of compliance

Compliance with PPE use increased significantly during the COVID-19 pandemic [13,19,21,22]. However, in the post-COVID-19 era, the compliance of our HCWs toward PPE use needs to be improved at certain points, which may need education intervention. The compliance of our HCWs post-COVID-19 era did not differ from the previous reports. The overall mean compliance score was 26.6 out of 40 points. Stratifying scores to levels, only 12.6 were considered good compliance; most were considered moderate levels (83.2%), while 4.2% were deemed poor. Of all compliance items, scores were higher in the importance of education and training to maintain good compliance, awareness of positive feedback from high-performing staff, and awareness of the corrective action for non-complying staff. This does not agree with the study done in Qatar [19]. Better compliance rates can be seen among Qatari HCWs. 44.1% were in full compliance with infection prevention and control measures. The highest compliance rate was recorded in Egypt [20], with most nurses (81.9%) adhering to the use of PPE, HH, and IPC measures. It is necessary to improve compliance toward PPE use. Continuous education and training among healthcare professionals could boost compliance with PPE use. In addition, strengthening policies and procedures and the availability of PPE may result in better compliance rates among hospital staff, particularly in critical care units within healthcare settings.

Significant factor of compliance

Increasing age, non-Bahrainis, and increasing years of experience were the factors associated with increased compliance. Interestingly, doctors by profession exhibited significantly lower perception and compliance scores than the other HCWs (p<0.05). Furthermore, our post-hoc analysis determined that doctors seem to have the lowest scores in perception and compliance compared to the nurses and physio/respiratory therapists. Several reasons could contribute to this effect, mainly due to a lack of staffing and work overload leading to a lack of participation in educational courses. This contradicted the reports of El-Sokkary et al. (2021) [13]. Better compliance with appropriate PPE practices was seen in female physicians' medical specialties with fewer years of working experience and working more than eight hours per day. In previous reports done in the USA [5], according to multivariate analysis, increased non-adherence with all five components of the contact isolation precaution was revealed, and non-compliance with HH before wearing gowns and gloves was associated with increasing burden of isolation. However, in Nigeria [17], age, gender, profession, and qualification did not significantly influence HCWs' access to PPE. In our study, however, gender and having received PPE education during college and previous hospital employment were not associated with compliance with PPE use, which was consistent with previous reports. Not opposing these reports, two fundamental themes emerged based on a quality study by Dimitriadou et al. (2022), such as infection prevention measures in practice and factors affecting compliance with infection prevention measures [16].

This study revealed that the correlation between perception and compliance scores was positively statistically significant (p<0.001), which suggests that the increase in the score of compliance correlates with the increase in the score of perception. This is almost consistent with the study of Sowar et al. (2023), indicating a positive correlation between perception and knowledge scores toward HH. More investigations are warranted to determine the link between perception and compliance toward PPE use.

Participation in courses and training

Participation in courses and training could lead to a better perspective on PPE use among HCWs. In this study, nearly all HCWs (95.8%) received education about the use of PPE, and a vast majority (77.2%) attended PPE courses or training in GH. Our data further indicates that education received within GHs significantly influences compliance scores (p=0.0021). Several studies documented that participation in courses and training increased the knowledge and compliance of HCWs to a greater extent [12,13,17]. Hence, the provision of training and education are keys to bridging the gaps toward adherence to PPE use in critical care settings.

Barriers to PPE use

Full adherence to PPE use cannot be achieved without identifying and addressing the barriers. In this study, the belief that compliance with PPE could affect performing procedures, the belief that adherence to PPE use was affected by the practice of colleagues or professional groups, and the belief that wearing a gown and gloves each time entering a patient's room is a waste of resources were some of the most important barriers affecting compliance to PPE use. Notwithstanding these reports, a review of 56 papers found that the most frequently mentioned barriers to complying with PPE recommendations include PPE availability, discomfort, inconvenience, perceived difficulty and effectiveness, and adverse impact on patient care [15]. However, in Nigeria [17], the study documented insufficient funds to buy PPEs, inaccessibility of PPE, and inadequate training on how to use PPE were the most prominent barriers to adherence to infection control policies. This study emphasized the importance of addressing these barriers to improve compliance and prevention rates in a healthcare environment.

Limitations

This study is bound to some limitations. First, the sample size is inadequate (N=119), limiting the generalizability of the findings. The non-responders also contributed to this effect and could be a source of potential bias; hence, this study's sample may not represent a true population. Second, the convenience sampling method could result in sampling bias and lead to a lack of diversity in the target population. Third, most HCWs were nurses; hence, comparing job categories was inconclusive. Lastly, being a cross-sectional survey necessitates a large sample size to represent the study population precisely; however, this was not reflected in our population due to the small size involved.

Recommendations

This study recommends several methods to increase compliance with PPE use. First, reducing workload may reduce HCWs' stress and increase compliance toward PPE use. Second, policies and procedures for strict compliance with wearing PPE should be strengthened. Third, more courses and training about the proper use of PPE should be advocated, particularly among doctors assigned to critical care units. Fourth, a multi-center study approach is recommended, probably at a national level, involving a bigger sample size that could provide a better view of HCWs' perception and compliance toward PPE in critical care settings. In addition, we recommend using multi-center or stratified sampling methods for future studies to help overcome limitations encountered in this study.

## Conclusions

The perception of HCWs toward PPE use achieved satisfactory ratings, but their actual compliance needs more improvement. Being older, non-Bahrainis, and having more years of experience tended to demonstrate better perception and compliance toward PPE use. Interestingly, doctors' perceptions of and compliance with PPE practices were lower than those of other HCWs. It is important to highlight that most of the HCWs working in critical care settings had attended education about the appropriate use of PPE. There's a need to improve PPE compliance among healthcare workers, particularly among doctors in GHs, Bahrain. Strengthening policies and procedures could achieve a better compliance rate among healthcare staff. Further, the provision of PPE training and education, the availability of PPE, and a healthy workplace environment are keys to improving adherence to PPE use.

## Appendices

Appendix 1

**Table 5:** Assessment of perception about PPE use. PPE: personal protective equipment. Adapted from El-Sokarry et al. (2021) [13]; Dimitriadou et al. (2022) [16]; Abed Alah et al. (2021) [18]. Note: Consent was obtained from the original publisher.

Perception						
No.	Criteria	Response				
		Strongly disagree	Disagree	Neutral	Agree	Strongly agree
1	Do you think that wearing PPEs is helping in preventing health care-associated infection (HAI)?					
2	Do you think that proper sequence of donning and doffing of PPEs can make difference on preventing HAI?					
3	Do you think that wearing gown and gloves each time entering patient room is a waste of resources?					
5	Do you feel that there is a good infection control practices in your department (example: HH, PPE usage, standard precautions, etc?)					
6	Do you think that you still need to wear PPE with or without procedure whenever you enter the patient room?					
7	Do wearing PPEs gives you discomfort while performing procedures on patients?					
8	Do you think wearing PPE requires a lot of your time and effort?					

Appendix 2 

**Table 6:** Assessment of compliance about PPE use. PPE: personal protective equipment, IC: infection control. Adapted from El-Sokarry et al. (2021) [13]; Dimitriadou et al. (2022) [16]; Abed Alah et al. (2021) [18]. Note: Consent was obtained from the original publisher.

Compliance questions						
No.	Criteria	Response				
		Strongly disagree	Disagree	Neutral	Agree	Strongly agree
1	Dose regular education and training on PPEs help you to maintain good compliance?					
2	Are you aware of any positive feedback for those high-performing staff when it comes to IC practices compliance?					
3	Are you aware of any corrective action for non-complying staff?					
4	Do workload or stressful situations affect your compliance on PPE use?					
5	Do you think compliance to PPE affect your success in preforming procedure?					
6	Do shortages on PPE supplies affect your motivation to comply?					
7	Was your adherence to PPE use influenced by the presence of supervisors or infection control staff/other auditor?					
8	Was your adherence to PPE use affected by the practice of your colleagues or other professional groups (i.e. Drs, RT, etc.)?					
